# Effect of physical exercise on the hippocampus and global grey matter volume in breast cancer patients: A randomized controlled trial (PAM study)

**DOI:** 10.1016/j.nicl.2022.103292

**Published:** 2022-12-14

**Authors:** E.W. Koevoets, M.I. Geerlings, E.M. Monninkhof, R. Mandl, L. Witlox, E. van der Wall, M.M. Stuiver, G.S. Sonke, M.J. Velthuis, J.J. Jobsen, J. van der Palen, M.E.M.M. Bos, E. Göker, M.B.E. Menke-Pluijmers, D.W. Sommeijer, A.M. May, on behalf of PAM study group, Annebeth W. Haringhuizen, Wim A. van der Steeg, Frederiek Terheggen, Charlotte Blanken-Peeters, Harold Fliervoet, Margrethe S. Schlooz-Vries, Tanja G. Frakking, Marc W.A. van Tilburg, Corina Oldenhuis, Maartje F. Sier, Carmen C. van der Pol, Lidwine W. Tick, Nel A. van Holsteijn, M.B. de Ruiter, S.B. Schagen

**Affiliations:** a Department of Medical Oncology, Ziekenhuis Gelderse Vallei, Ede, the Netherlands; b Isala Hospital, Zwolle, the Netherlands; c Department of Medical Oncology, Bravis Hospital, Roosendaal, the Netherlands; d Department of Oncological Surgery, Rijnstate Hospital, Arnhem, the Netherlands; e Department of Oncology, Canisius Wilhelmina Hospital, Nijmegen, the Netherlands; f Department of Surgical Oncology, Radboud University Medical Center, Nijmegen, the Netherlands; g Beatrix Hospital, Rivas Health Care Centre, Gorinchem, the Netherlands; h Department of Surgery, Hospital St Jansdal, Harderwijk, the Netherlands; i Department of Internal Medicine, Treant Zorggroep, Emmen, the Netherlands; j Department of Surgery, Ziekenhuis Rivierenland, Tiel, the Netherlands; k Department of Surgery, St Antonius Hospital, Nieuwegein, the Netherlands; l Department of Surgery, Alrijne Ziekenhuis, Leiderdorp, the Netherlands; m Department of Internal Medicine, Máxima Medisch Centrum, Eindhoven, the Netherlands; n Breast Cancer Center, Reinier de Graaf Hospital, Delft, the Netherlands; a Julius Center for Health Sciences and Primary Care, University Medical Center Utrecht and Utrecht University, Utrecht, the Netherlands; b Division of Psychosocial Research and Epidemiology, Netherlands Cancer Institute, Amsterdam, the Netherlands; c Department of General Practice, Amsterdam UMC, Amsterdam, the Netherlands; d Department of Psychiatry, University Medical Center Utrecht and Utrecht University, Utrecht, the Netherlands; e Department of Medical Oncology, University Medical Center Utrecht, Utrecht University, Utrecht, the Netherlands; f Center for Quality of Life, Netherlands Cancer Institute, Amsterdam, the Netherlands; g Center of Expertise Urban Vitality, Faculty of Health, University of Applied Sciences, Amsterdam, the Netherlands; h Department of Medical Oncology, Netherlands Cancer Institute, Amsterdam, the Netherlands; i Netherlands Comprehensive Cancer Organisation (IKNL), Utrecht, the Netherlands; j Medical School Twente, Medisch Spectrum Twente, Enschede, the Netherlands; k Department of Research Methodology, Measurement, Universiteit Twente, Enschede, the Netherlands; l Department of Medical Oncology, ErasmusMC Cancer Institute, Rotterdam, the Netherlands; m Department of Medical Oncology, Alexander Monro Hospital, Bilthoven, the Netherlands; n Breast Clinic, Albert Schweitzer Hospital, Dordrecht, the Netherlands; o Department of Internal Medicine, Flevohospital, Almere, the Netherlands; p Department of Medical Oncology, Amsterdam UMC, Amsterdam, the Netherlands; q Brain and Cognition Group, University of Amsterdam, Amsterdam, the Netherlands

**Keywords:** Exercise, Hippocampus, Grey matter volume, Cortical thickness, Breast cancer, Fatigue, ACS, Amsterdam Cognition Scan, BMI, body mass index, CA, cornu ammonis, CAT12, Computational Anatomy Toolbox, CPET, cardiopulmonary exercise test, EORTC QLQ-C30, European Organisation for Research and Treatment of Cancer Quality of Life Questionnaire, ES, effect size, FWE, family-wise error, FWHM, full width at half maximum, GC-ML-DG, granule cell layer of dentate gyrus, HVLT-R total recall, Hopkins Verbal Learning Test-Revised total recall score, MNI, Montreal Neurological Institute, PAM, Physical Activity and Memory, RM, repetition maximum, SPM, Statistical Parameter Mapping, TIV, total intracranial volume, UMC, University Medical Center, VBM, voxel-based morphometry, VO_2peak_, relative maximum oxygen uptake

## Abstract

**Background:**

Physical exercise in cancer patients is a promising intervention to improve cognition and increase brain volume, including hippocampal volume. We investigated whether a 6-month exercise intervention primarily impacts total hippocampal volume and additionally hippocampal subfield volumes, cortical thickness and grey matter volume in previously physically inactive breast cancer patients. Furthermore, we evaluated associations with verbal memory.

**Methods:**

Chemotherapy-exposed breast cancer patients (stage I-III, 2–4 years post diagnosis) with cognitive problems were included and randomized in an exercise intervention (n = 70, age = 52.5 ± 9.0 years) or control group (n = 72, age = 53.2 ± 8.6 years). The intervention consisted of 2x1 hours/week of supervised aerobic and strength training and 2x1 hours/week Nordic or power walking. At baseline and at 6-month follow-up, volumetric brain measures were derived from 3D T1-weighted 3T magnetic resonance imaging scans, including hippocampal (subfield) volume (FreeSurfer), cortical thickness (CAT12), and grey matter volume (voxel-based morphometry CAT12). Physical fitness was measured with a cardiopulmonary exercise test. Memory functioning was measured with the Hopkins Verbal Learning Test-Revised (HVLT-R total recall) and Wordlist Learning of an online cognitive test battery, the Amsterdam Cognition Scan (ACS Wordlist Learning). An explorative analysis was conducted in highly fatigued patients (score of ≥ 39 on the symptom scale ‘fatigue’ of the European Organisation for Research and Treatment of Cancer Quality of Life Questionnaire), as previous research in this dataset has shown that the intervention improved cognition only in these patients.

**Results:**

Multiple regression analyses and voxel-based morphometry revealed no significant intervention effects on brain volume, although at baseline increased physical fitness was significantly related to larger brain volume (e.g., total hippocampal volume: R = 0.32, B = 21.7 mm^3^, 95 % CI = 3.0 – 40.4). Subgroup analyses showed an intervention effect in highly fatigued patients. Unexpectedly, these patients had significant reductions in hippocampal volume, compared to the control group (e.g., total hippocampal volume: B = −52.3 mm^3^, 95 % CI = −100.3 – −4.4)), which was related to improved memory functioning (HVLT-R total recall: B = −0.022, 95 % CI = −0.039 – −0.005; ACS Wordlist Learning: B = −0.039, 95 % CI = −0.062 – −0.015).

**Conclusions:**

No exercise intervention effects were found on hippocampal volume, hippocampal subfield volumes, cortical thickness or grey matter volume for the entire intervention group. Contrary to what we expected, in highly fatigued patients a reduction in hippocampal volume was found after the intervention, which was related to improved memory functioning. These results suggest that physical fitness may benefit cognition in specific groups and stress the importance of further research into the biological basis of this finding.

## Highlights

• No effect of 6 months exercise on brain volume in breast cancer patients.
• Positive relation between physical fitness and brain (volume) at baseline.
• A decrease in hippocampal volume post intervention in highly fatigued patients.
• Effects on volume, related to improved memory functioning, in highly fatigued women.
• Exercise is important for brain health and is more profitable for some patients.

## 1 Background

An increasing number of patients with breast cancer are facing late effects of cancer and cancer treatment (Dafni et al., 2019), among others cognitive decline. This is observed in a substantial subgroup of patients, particularly after treatment with chemotherapy. Incidence rates after diagnosis and treatment are estimated at approximately 25 % (Dijkshoorn et al., 2021). Multiple cognitive domains are affected, including learning and memory, attention, speed of information processing and executive functioning (Wefel et al., 2015). These cognitive problems are typically of a mild to moderate nature, but nevertheless may profoundly affect quality of life (Ahles and Root, 2018, Lange et al., 2019, Wefel et al., 2015). Therefore, interventions targeting these cognitive problems are needed and physical exercise is considered a promising intervention.

Studies have shown that brain alterations occur after treatment with chemotherapy, including global and local grey matter volumetric reductions (Amidi and Wu, 2019, Li and Caeyenberghs, 2018, McDonald, 2021). The hippocampus, essential for learning and memory functioning, might be particularly vulnerable and several cross-sectional and longitudinal neuroimaging studies observed smaller hippocampal volumes in chemotherapy-exposed breast cancer patients than in controls, which was in addition related to poorer memory function (Peukert et al., 2020). Animal studies have shown that this volume reduction might be explained by a significant decline in hippocampal neurogenesis (Sekeres et al., 2021).

Several randomized exercise intervention studies, with an intervention duration of 6 months or longer, have shown positive effects on cognitive functioning in healthy elderly (Biazus-Sehn et al., 2020, Falck et al., 2019), and in patients with mild cognitive impairment (Loprinzi et al., 2019). Additionally, volume increase or maintenance due to physical exercise has been found in the hippocampus (e.g., (Erickson et al., 2011, Ten Brinke et al., 2015)), with one of the proposed mechanisms being neuronal change and growth of new neurons, including the process of neurogenesis (van Praag, 2008). Also, increase in brain volume and reduced brain atrophy have been reported (Chen et al., 2020).

In a cross-sectional study conducted in breast cancer patients after completion of treatment, an association was found between higher cardiorespiratory fitness and larger hippocampal volume (Chaddock-Heyman et al., 2015). More specifically, hippocampal volume of relatively fit cancer survivors was comparable to hippocampal volume of non-cancer controls. However, since intervention studies with a prospective study design are missing in breast cancer patients, the effects of a physical exercise intervention on brain structure, including hippocampal volume effects, remain unknown.

The purpose of the ‘Physical Activity and Memory (PAM) study’ was to investigate the effects of a 6-month physical exercise intervention on cognitive functioning and brain structure, in physically inactive, chemotherapy-exposed women with breast cancer and cognitive problems. Earlier, we reported no positive effects of physical exercise on tested cognitive functioning, but beneficial effects on subjective cognitive functioning, fatigue, depression and quality of life (Koevoets et al., 2022). In a subgroup of highly fatigued patients, we did find improvements in tested cognition, in the domains of processing speed, and learning and memory functioning. Effects of physical exercise on hippocampal volume might be an explanation for this observation. Therefore, in the current study, we investigated the effects of the intervention on changes in hippocampal volume, our primary outcome measure. Hippocampal subfields, including the dentate gyrus, the site where adult human neurogenesis is believed to occur, and whole brain volume changes, were also investigated (Ming and Song, 2005). Additionally, we evaluated baseline associations between age, physical fitness and brain volume. Finally, in an exploratory analysis, we investigated the effects of the exercise intervention on brain volume in highly fatigued patients and the potential relation between hippocampal (subfield) volume and memory functioning.

## 2 Methods

### 2.1 Design

The PAM study is an adequately powered two-armed multi-center phase III randomized controlled exercise trial in women with breast cancer who were physically inactive and had cognitive problems. Data were collected between December 2016 and September 2020 at the University Medical Center (UMC) Utrecht (Utrecht, the Netherlands) at baseline (before randomization) and follow-up (after 6 months). All study procedures were approved by the Medical Ethics Committee of the UMC Utrecht and all patients provided written informed consent.

Recruitment, study design and effects of the intervention on cognitive functioning and patient-reported outcomes have been described in detail elsewhere (Koevoets et al., 2022, Witlox et al., 2019).

### 2.2 Patients

Eligible for inclusion were women, diagnosed with stage I-III breast cancer 2–4 years before study enrollment, who had been treated with (neo)adjuvant chemotherapy, were between 30 and 75 years, reported to be relatively inactive (150 min or less of moderate to vigorous physical activity per week), were sufficiently proficient in the Dutch language, and agreed to be randomized to either the intervention or control group. Only patients who reported cognitive complaints since cancer diagnosis or treatment (in a semi-structured interview) and who demonstrated lower than expected cognitive performance on onlineneuropsychological testing were included (≥ 1 normative standard deviation (based on sex and age) lower on ≥ 2 tests in ≥ 2 (out of 5) cognitive domains) (Table 1) (Feenstra et al., 2018, Koevoets et al., 2022, Witlox et al., 2019). Exclusion criteria were known neurological disorders that may affect cognition (e.g., dementia, multiple sclerosis), contraindications for MRI scanning or exercise, and planned switching or stopping endocrine therapy four months prior to study enrolment or during the study period.

**Table 1:** Content of the Amsterdam Cognition Scan.

Test domain	Online test	Main outcome measures	Traditional equivalent
**Learning and memory**	Wordlist Learning	Total number of correct responses (Learning: trials 1–5)	Dutch version of Rey Auditory Verbal Learning Test (immediate recall, delayed recall and recognition)
	Wordlist Delayed Recall Wordlist Recognition		
**Attention and working memory**	Box Tapping	Total number of correctly repeated sequences	Corsi Block-Tapping Test


	Digit Sequences I		WAIS-III Digit Span forward
	Digit Sequences II		WAIS-III Digit Span backward
**Processing speed**	Reaction Time	Mean reaction time (ms)	Visual Reaction Time (subtest FePsy)
	Connecting the Dots I	Completion time (s)	Trail Making Test A
**Executive functioning**	Connecting the Dots II	Completion time (s)	Trail Making Test B
	Place the Beads	Total number of extra moves	Tower of London, Drexel University (ToL-dx)
**Motor functioning**	Fill the Grid	Completion time (s)	Grooved Pegboard

Age, education, menopausal status, and age at menopause were assessed with a questionnaire. Clinical characteristics were retrieved form medical records. Medical use (including endocrine therapy use) was assessed during an interview.

### 2.3 Recruitment and randomization

**Fig. 1:**
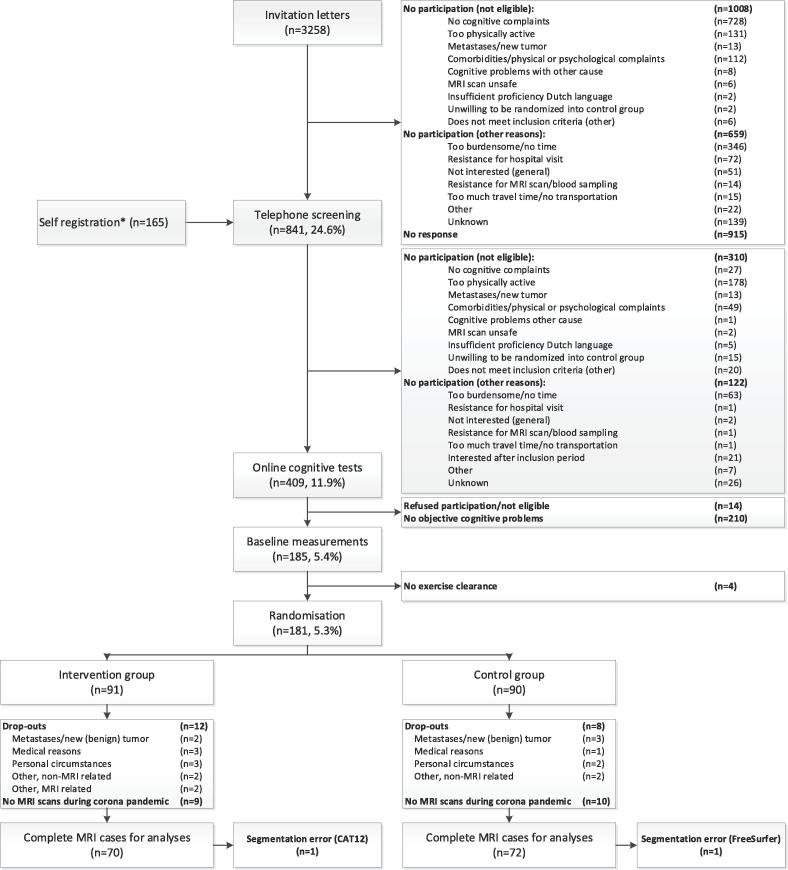
Flowchart of inclusion and randomization procedures of the Physical Activity and Memory (PAM) study patients. * Information through social media, pamphlets, and by word of mouth.

Patients were recruited through invitation letters sent from 21 Dutch hospitals (n = 3,258) or self-registration (n = 165) (Fig. 1). Patients were screened for eligibility through telephone screening (n = 841) and subsequently completed the Amsterdam Cognition Scan (ACS) for confirmation of affected cognitive functioning (n = 409) (Table 1). After baseline measurements, patients were randomized to the intervention or control group (1:1) by a study team member using a computer-generated sequence ensuring blinded treatment allocation, which was provided by the data-management department (UMC Utrecht). Randomization was stratified by age category (30–44, 45–59, 60–75 years) and endocrine therapy (yes/no).

### 2.4 Exercise intervention

The physical exercise intervention comprised 6 months of twice a week one hour supervised training sessions, consisting of both aerobic training and strength exercises, and twice a week one hour Nordic or power walking sessions. Supervised sessions were delivered by a physiotherapist, close to the patient’s home address. Intensity of the aerobic training was based on individual physical fitness levels, which were measured at baseline with a cardiopulmonary exercise test (CPET); intensity for strength exercises was based on repeated 20-repetition maximum (20-RM) and 15-RM muscle strength tests, performed by the physiotherapist. Intensity progressed throughout the supervised program (Table 2). For the Nordic or power walking program, intensity was set at baseline, at 55 % - 65 % of the heart rate reserve. Heart rate monitors were provided by the study team. After one month of training, a member of the study team verified protocol adherence during a monitoring visit.

### 2.5 Control group

Women in the (waiting list) control group were asked to maintain their usual physical activity levels and were offered a shortened exercise program of 12 weeks, after study completion.

**Table 2:** Supervised exercise program of the PAM study.

Week	Aerobic	Strength
**1**–**4**	40 % - 60 % HRR	One circuit of 20–25 repetitions. Weights based on 20-RM tests (repeated every 4 weeks).
**5**–**9**	60 % - 70 % HRR 15–20 min, plus 70 % - 89 % HRR 5–10 min	Exercises: legs (squat, lunges, calve raises), arms (biceps curl, triceps extension), shoulder (shoulder press), thorax (Barbell bench press), back (rowing). Abdomen: crunch 30–40 repetitions.
**10**–**17**	Interval training: 10x30 s vigorous to maximal exercise, alternated with 1 min active rest, plus 10 min 60 % - 75 % endurance	Two circuits of 15–20 repetitions. Weights based on 15-RM tests (repeated every 4 weeks).
**18**–**26**	Interval training: 2 circuits of 8x30 s vigorous to maximal exercise, alternated with 1 min active rest, plus 5 min 60 % - 75 % endurance	Exercises: legs (squat), arms (biceps curl, triceps extension), shoulder (shoulder press), thorax (Barbell bench press), back (rowing). Abdomen: crunch 30–40 repetitions; hoover/planking 2x 45 s.

Abbreviations: PAM, Physical Activity and Memory; HRR, heart rate reserve; RM, repetition maximum.

### 2.6 MRI acquisition and processing

Structural brain measures were derived from a 3.0 Tesla Philips Achieva full-body MRI scanner with a 8-channel head coil at baseline and after 6 months. All scans were acquired on the same scanner with a standardized protocol that included a sagittal 3D T1-weighted sequence (TR/TE = 7.9/4.5 ms; 192 slices; FOV(mm) = 256x232; 1.00 mm isotropic voxels). Scan quality was visually checked.

#### 2.6.1 Hippocampal segmentation

Automatic hippocampal (subfield) segmentations were performed using FreeSurfer image analysis (version 7.1.1; Boston, MA, USA; https://surfer.nmr.mgh.harvard.edu/ (Fischl, 2012)). The recon-all script for the longitudinal pipeline was used (Reuter et al., 2012).

Total hippocampal volume, our primary outcome measure, was calculated by the hippocampal subfield segmentation module. Additionally, this module calculated 12 hippocampal subfields of which 6 subfields were pre-selected for analyses to reduce false positive findings, based on relevance and accuracy (Van Leemput et al., 2009): CA1, CA2/3, CA4, subiculum, tail and the granular cells layer of the dentate gyrus. Volume estimates for total hippocampal volume and hippocampal subfields were analyzed for left and right hemispheres separately. All scans were inspected by a neuroradiologist for (clinically relevant) incidental findings. Hippocampal (subfield) segmentation data of patients with the highest and lowest hippocampal subfield volumes at either measurement, or largest changes between measurements, were selected and visually checked for segmentation errors with the FreeSurfer image viewer, Freeview (n = 40 patients). Furthermore, segmentations of patients with (clinically irrelevant) findings reported by the neuroradiologist, such as small cysts and small meningiomas, were visually checked (n = 17 patients).

#### 2.6.2 Global grey matter changes

Global cortical thickness volume was generated by the longitudinal pipeline of FreeSurfer v.7.1.1. (https://surfer.nmr.mgh.harvard.edu/ (Reuter et al., 2012)).

Voxel-based morphometry (VBM) was used to assess grey matter volume changes. We used the ‘longitudinal model for small changes’ of the Computational Anatomy Toolbox (CAT12) (https://www.neuro.uni-jena.de/cat/), based on Statistical Parameter Mapping (SPM12) software version 7771 (https://www.fil.ion.ucl.ac.uk/spm/) running under MATLAB version R2018a, to implement VBM methods. Preprocessing steps according to the CAT12 manual were followed in detail (Gaser and Dahnke, 2016). In brief, 3D T1-weighted MRI scans were modulated, normalized to Montreal Neurological Institute (MNI) space and segmented into white matter, grey matter and cerebrospinal fluid. Total intracranial volume (TIV) was estimated by adding white matter, grey matter and cerebrospinal fluid volume. Then, we used quality assurance tests in CAT12 to check noise, bias, and weighted average ratings of image and processing quality. Scans with ratings below ‘B’ (‘B-‘: n = 4; ‘C+’: n = 1) were closely monitored throughout the process, and were deemed of high enough quality for inclusion. After this quality check, data was smoothed with a 6 mm Gaussian FWHM kernel. The normalized, smoothed grey matter maps were used for statistical parametric mapping.

### 2.7 Other outcomes

#### 2.7.1 Physical fitness

Physical fitness was measured with a maximal cycle CPET using a ramp protocol, including continuous breathing gas analysis and electrocardiogram monitoring. Over the final 30 s of exercise, we calculated an average for relative maximum oxygen uptake (VO_2peak_), divided by baseline body weight (in kg).

#### 2.7.2 Memory functioning

Cognitive functioning was primarily measured with the total recall score of the Hopkins Verbal Learning Test-Revised (HVLT-R total recall) (Benedict et al., 1998), the gold standard for memory functioning in neuro(oncological) trials. Additionally, the Wordlist Learning trial of the ACS is included (ACS Wordlist Learning) (Table 1), an outcome for learning and memory functioning on which a significant effect of the exercise intervention in highly fatigued patients was observed.

More detailed information regarding these outcomes and additional cognitive outcomes are described elsewhere (Koevoets et al., 2022, Witlox et al., 2019).

#### 2.7.3 Fatigue

To identify highly fatigued patients, we calculated the patients’ score on the symptom scale ‘fatigue’ of the European Organisation for Research and Treatment of Cancer Quality of Life Questionnaire (EORTC QLQ-C30), which ranged from 0 to 100 (Aaronson et al., 1993). A higher score represented higher symptom burden and patients with a score of ≥ 39 were considered highly fatigued (Giesinger et al., 2020).

### 2.8 Statistical analyses

#### 2.8.1 General

For all analyses, the intention-to-treat principle was applied with complete MRI case data. Total hippocampal volume was our primary MRI outcome and other analyses were explorative. Hippocampal (subfield) volume, cortical thickness and baseline association analyses were performed with IBM SPSS Statistics for Windows version 25.0.0.2 (IBM Corp, 2017). Critical two-sided alpha value was set for all analyses at 0.05. Statistically significant voxels were derived with SPM12, with an initial threshold of 0.001, and then considered significant at peak level with a threshold of 0.05, family-wise error (FWE)-corrected.

#### 2.8.2 Baseline associations and relations over time

To verify the expected inverse association between age and brain volume, we performed simple regression analyses between age and total hippocampal volume, cortical thickness, and total grey matter volume at baseline. Differences within the total group were investigated to determine whether total hippocampal volume, cortical thickness, and total grey matter volume changed over a time period of 6 months. We executed repeated measures analysis of variance including baseline and follow-up measurement of total hippocampal volume, cortical thickness, and total grey matter volume. Additionally, we used a multiple regression analysis to examine the association at baseline between physical fitness and total hippocampal volume, cortical thickness, and total grey matter volume, with age as covariate.

#### 2.8.3 Intervention effects

First, we assessed intervention effects on physical fitness in the sample used in this paper. We executed a multiple regression analysis, with VO_2peak_ as dependent variable, and with baseline VO_2peak_ and stratification factors (age, endocrine treatment) as covariates. To assess between-group differences on hippocampal volume measures, we used multiple regression analyses with total hippocampal volume or subfield hippocampal volume at follow-up as the dependent variable, and baseline values of total hippocampal volume or subfield hippocampal volume, stratification factors, and total intracranial volume (TIV), which was estimated by CAT12 and averaged over the two measurements, as covariates.

Whole brain approaches included average cortical thickness, total grey matter volume, and VBM cluster analyses. Cortical thickness and total grey matter volume were analyzed with multiple regression analyses with cortical thickness or total grey matter volume at follow-up as the dependent variable, and baseline measurement of cortical thickness or total grey matter volume, age, endocrine treatment, and TIV as covariates. For VBM analyses, a basic model for two group longitudinal data was built for the smoothed grey matter segments, where we investigated group-by-time interaction and main effects (CAT12 manual: (Gaser and Dahnke, 2016)). Threshold of implicit masking was set at 0.1.

As per protocol analyses, all analyses were repeated for patients with a minimal attendance to the exercise intervention of 80 %, which was calculated separately for the supervised exercise program and Nordic/power walking (number of attended sessions / number of offered sessions * 100) and averaged.

#### 2.8.4 Highly fatigued patients

To assess intervention effects on physical fitness in highly fatigued patients, the multiple regression analysis mentioned in 2.8.3 was repeated in this sample. Furthermore, in the subsample of highly fatigued patients, exercise effects on total hippocampal volume, hippocampal subfield volumes, cortical thickness, and total grey matter volume were investigated using multiple regression analyses with baseline measurement of the abovementioned variables, age, endocrine treatment, and TIV as covariates in the first model. As this subsample is not randomized, we decided to add covariates that represent potential differences in demographic variables between the intervention and control group and which could influence the interpretation of the outcomes in a second model. Therefore, body mass index (BMI) at baseline and educational level were added to the covariates included in the first model. Within-group analyses, using repeated measures analysis of variance with both measurements, were executed to investigate whether significant between-group differences were mainly driven by intervention effects or differences between baseline and follow-up in the control group. Furthermore, VBM analyses were repeated in this sample.

If a significant effect of the intervention on hippocampal (subfield) volumes is observed, the difference in hippocampal (subfield) volume as a potential predictor of the difference on HVLT-R total recall, the primary outcome measure of the PAM trial, is investigated, to assess whether this might explain the reduction in memory problems in this group. Multiple regression analyses were executed, including baseline HVLT-R total recall and group as covariate. Similar analyses were done for ACS Wordlist Learning.

## 3 Results

### 3.1 Patient characteristics

In the PAM study, we randomized 181 patients to the intervention (*n =* 91) or control group (*n =* 90). For 70 patients in the intervention group (mean age = 52.5 ± 9.0 years) and 72 patients in the control group (mean age = 53.2 ± 8.6 years), MRI data were available at baseline and 6-month follow-up (Fig. 1/Table 3). Groups were comparable on all demographic and clinical characteristics, except for psychotropic medication use (Table 3). Drop-outs were caused primarily due to inaccessibility of the MRI scanner during the corona pandemic (n = 19). Other reasons for drop-out were (possible) metastases/new (benign) tumor (n = 5), personal circumstances (n = 5), medical reasons (n = 3), or other (n = 7). Data of 1 control patient was missing due to segmentation errors. For VBM analyses in CAT12, segmentation of 1 additional intervention patient’s data failed, probably because of enlarged ventricles. Drop-outs had a lower age at menopause (drop-outs: 44.9 ± 5.6 years vs inclusions: 47.8 ± 5.9 years), received more often a combination of neoadjuvant and adjuvant chemotherapy, and were included closer to chemotherapy completion (drop-outs: 2.4 ± 0.6 years vs inclusions: 2.7 ± 0.6 years). No other differences were observed.

**Table 3:** Baseline demographic and treatment characteristics.

	Intervention group *(n = 70)*	Control group *(n = 72)*	p
**Age (years)**	52.5 (9.0)	53.2 (8.6)	0.550
**Education level (n (%))**			
**High**	34 (48.6)	30 (41.7)	
**Middle**	36 (51.4)	42 (58.3)	
**Low**	0 (0)	0 (0)	0.451
**Physical fitness (VO_2peak_ in ml/min/kg)**	23.5 (4.6)	24.8 (6.1)	0.175
Body mass index (BMI)	29.6 (5.8)	28.3 (5.4)	0.197
**Menopausal status (n (%))**			
**Pre/*peri***	9 (12.9)	7 (9.7)	
**Post**	61 (87.1)	65 (90.3)	0.575
**Age of menopause (years)**	48.0 (6.4)	47.7 (5.4)	0.917
Time since diagnosis (years)*	3.1 (0.7)	3.1 (0.6)	1.000
**Tumor grade (n (%))**			
**I**	11 (15.7)	5 (6.9)	
**II**	28 (40.0)	31 (43.1)	
**III**	24 (34.3)	28 (38.9)	
**Unknown**	7 (10.0)	8 (11.1)	0.447
**Surgery (n (%))**	70 (1 0 0)	72 (1 0 0)	1.000
**Chemotherapy timing (n (%))**			
**Neoadjuvant**	31 (44.3)	34 (47.2)	
**Adjuvant**	37 (52.9)	36 (50.0)	
**Both**	1 (1.4)	1 (1.4)	
**Unknown**	1 (1.4)	1 (1.4)	0.980
Time since completion chemotherapy (years)*	2.7 (0.7)	2.7 (0.6)	0.625
**Radiation (n (%))**			
**Yes**	54 (77.1)	55 (76.4)	
**No**	16 (22.9)	17 (23.6)	0.964
**Targeted therapy (n (%))**			
**Yes**	16 (22.9)	15 (20.8)	
**No**	54 (77.1)	56 (77.8)	
**Unknown**		1 (1.4)	0.559
**Endocrine therapy (n (%))**			
**Yes**	44 (62.9)	45 (62.5)	
**No**	26 (37.1)	27 (37.5)	0.914
**Medication use (n (%))**			
**Cardiovascular**	14 (20.0)	14 (19.4)	0.967
**Anti-diabetic**	1 (1.4)	1 (1.4)	0.992
**Psychotropic**	21 (30.0)	12 (16.7)	0.040*
**Pain medication**	11 (15.7)	12 (16.7)	0.971

Values indicate mean (SD) unless indicated otherwise. P values indicate overall group differences.

*For time since diagnosis, average years was based on 63 intervention patients and 67 control patients. For time since completion chemotherapy, average years was based on 65 intervention patients and 62 control patients.

An attendance rate of at least 80 % was reached by 74 % (*n =* 52) of the intervention patients (exercise supervised by physiotherapist: 79 %; Nordic/power walking: 69 %), with a median attendance of 88 % (range 0–100 %, mean = 81 % ± 21).

### 3.2 Baseline associations and relations over time

At baseline, total hippocampal volume (R = 0.26, B = −18.2 mm^3^, 95 % CI = −29.6 – −6.9), cortical thickness (R = 0.41, B = −0.0031 mm, 95 % CI = −0.0043 – −0.0020), and total grey matter volume (R = 0.40, B = −2.19 cm^3^, 95 % CI = −3.04 – −1.33) showed a statistically significant inverse association with age. However, within the total group, no differences were found for total hippocampal volume, cortical thickness, and total grey matter volume from baseline to 6-month follow-up (p > 0.05).

Physical fitness showed a statistically significant positive association with total hippocampal volume (R = 0.32, B = 21.7 mm^3^, 95 % CI = 3.0 – 40.4), cortical thickness (R = 0.45, B = 0.0023 mm, 95 % CI = 0.0004 – 0.0042), and total grey matter volume at baseline (R = 0.47, B = 2.32 cm^3^, 95 % CI = 0.95 – 3.70), while adjusting for age.

### 3.3 Intervention effects

**Fig. 2:**
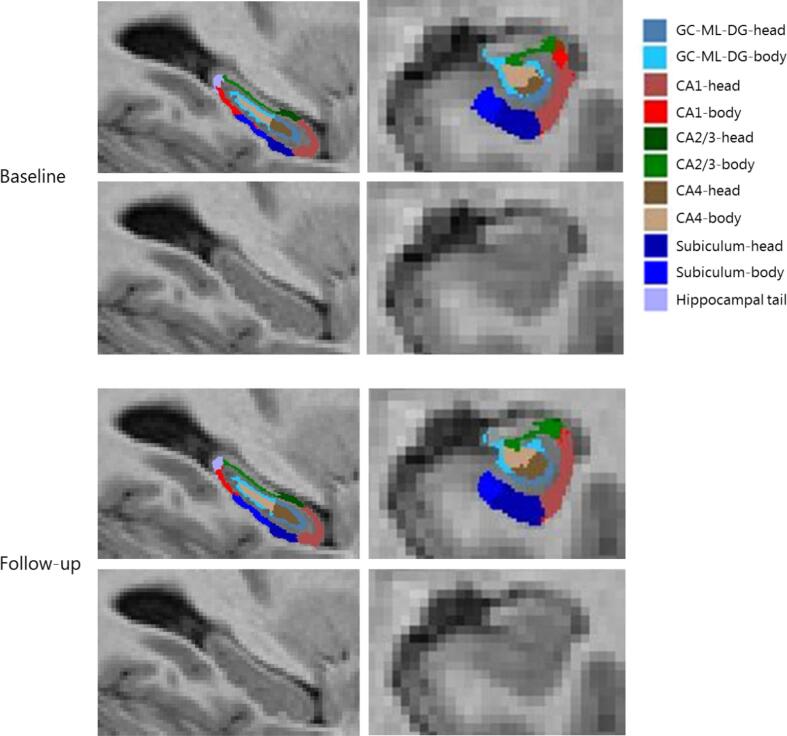
A representative example of the automated hippocampal subfield segmentation by FreeSurfer of one participant at baseline and follow-up. Abbreviations: GC-ML-DG, granule cell layer of dentate gyrus; CA, cornu ammonis.

Physical fitness improved after the intervention in comparison to the control group (B = 1.40 ml/min/kg, 95 % CI = 0.55 – 2.26, ES = 0.26). Total hippocampal volume at baseline was on average 6643 mm^3^ ± 623 mm^3^ for the intervention and 6623 mm^3^ ± 599 mm^3^ for the control group, and did not significantly change during the 6-month follow-up period in the intervention group, compared to the control group (B = −8.9 mm^3^, 95 % CI = −37.0 – 19.3) (Table 4). Additionally, exercise did not yield effects at hippocampal subfield level (Table 4). In Fig. 2, a representative example of the automated hippocampal subfield segmentation is presented.

**Table 4:** Intervention effects on hippocampal (subfield) volume, cortical thickness, and total grey matter volume.

Outcome measures		Intervention	Control	Treatment effect† (95 % CI)	Effect Size‡	% change intervention^#^	% change control^#^
**Total hippocampal volume**		*N = 70*	*N = 71*				
Total	Baseline	6643 (6 2 3)	6623 (5 9 9)				
	Follow-up	6640 (6 1 9)	6628 (6 0 4)	−8.9 (−37.0 – 19.3)	−0.01	−0.04	0.07
Left	Baseline	3290 (3 4 0)	3275 (3 1 4)				
	Follow-up	3289 (3 3 5)	3275 (3 1 8)	−1.5 (−20.0 – 17.0)	0.00	−0.02	0.00
Right	Baseline	3353 (3 0 8)	3348 (3 0 8)				
	Follow-up	3351 (3 0 9)	3353 (3 0 7)	−7.6 (−24.5 – 9.3)	−0.02	−0.06	0.14
**GC-ML-DG**							
Left	Baseline	270 (31)	266 (25)				
	Follow-up	268 (30)	267 (26)	−2.29 (−4.92 – 0.34)	−0.08	−0.45	0.38
Right	Baseline	278 (27)	280 (30)				
	Follow-up	278 (27)	280 (30)	−1.02 (−3.23 – 1.18)	−0.04	−0.22	0.02
**CA1**							
Left	Baseline	603 (76)	609 (71)				
	Follow-up	603 (75)	609 (74)	−0.55 (−4.77 – 3.66)	−0.01	0.01	0.07
Right	Baseline	637 (76)	645 (73)				
	Follow-up	638 (77)	647 (73)	−2.01 (−6.55 – 2.52)	−0.03	0.05	0.38
**CA2/3**							
Left	Baseline	201 (32)	194 (27)				
	Follow-up	200 (32)	195 (27)	−1.70 (−4.05 – 0.71)	−0.06	−0.59	0.32
Right	Baseline	222 (27)	224 (30)				
	Follow-up	222 (27)	224 (30)	−0.75 (−3.17 – 1.67)	−0.03	−0.09	0.17
**CA4**							
Left	Baseline	235 (27)	232 (22)				
	Follow-up	234 (27)	233 (23)	−1.59 (−4.05 – 0.87)	−0.06	−0.34	0.31
Right	Baseline	240 (24)	242 (28)				
	Follow-up	240 (23)	242 (27)	−0.67 (−2.72 – 1.39)	−0.03	−0.08	0.05
**Subiculum**							
Left	Baseline	406 (53)	412 (64)				
	Follow-up	407 (51)	411 (61)	1.53 (−2.48 – 5.55)	0.03	0.18	−0.30
Right	Baseline	388 (41)	386 (50)				
	Follow-up	387 (44)	387 (51)	−1.79 (−5.41 – 1.84)	−0.04	−0.26	0.21
**Hippocampal tail**							
Left	Baseline	556 (88)	541 (65)				
	Follow-up	558 (87)	540 (66)	2.66 (−2.85 – 8.16)	0.03	0.23	−0.19
Right	Baseline	579 (96)	562 (68)				
	Follow-up	580 (96)	562 (69)	0.81 (−3.70 – 5.32)	0.01	0.24	0.08
**Cortical thickness (in mm)^**							
	Baseline	2.41 (0.07)	2.41 (0.07)				
	Follow-up	2.41 (0.07)	2.41 (0.07)	−0.003 (−0.012 – 0.006)	−0.04	0.02	0.17
**Total grey matter (in cm^3^)^**							
	Baseline	601 (44)	599 (52)				
	Follow-up	601 (43)	599 (51)	−1.14 (−4.20 – 1.91)	−0.02	−0.13	0.06

Values indicate mean (SD) and are presented in mm^3^, unless denoted otherwise.

Abbreviations: GC-ML-DG, granule cell layer of dentate gyrus, CA, cornu ammonis.

† The intervention effect is the regression coefficient of a linear regression analysis adjusted for baseline, age, endocrine therapy and total intracranial volume calculated by CAT12.

‡ Effect Sizes (ES) were calculated by dividing Beta by the pooled SD at baseline, with positive ESs meaning a beneficial effect of the intervention on a specific outcome. ESs < 0.2 indicate “no difference”, ESs between 0.2 and 0.5 indicate “small differences”, ESs between 0.5 and 0.8 indicate “medium differences” and ESs ≥ 0.8 indicate “large differences” (Cohen, 2013). An ES of 0.5 or higher was considered clinically relevant (Norman et al., 2003).

^#^ Calculation for average % change between baseline and follow-up: (follow-up - baseline)/baseline * 100.

^ Data of one additional patient from the intervention group was missing due to segmentation errors.

At follow-up, cortical thickness did not significantly change in the intervention group, compared to the control group (B = −0.003 mm, 95 % CI = −0.012 – 0.006) (Table 4). Additionally, total grey matter volume did not significantly change in the intervention group, compared to the control group (B = −1.14 cm^3^, 95 % CI = −4.20 – 1.91) (Table 4).

FWE-corrected analyses on volume increase in the intervention group compared to the control group (or reversed), did not reveal significant clusters.

All per protocol analyses, using data of 52 patients in the intervention and all 72 patients in the control group, yielded comparable results (data shown in Additional file).

### 3.4 Highly fatigued patients

In highly fatigued patients, physical fitness improved in the intervention group, compared to the control group (B = 1.47 ml/min/kg, 95 % CI = 0.04 – 2.90). Moreover, compared to the control group, a decrease in total hippocampal volume (B = −52.3 mm^3^, 95 % CI = −100.3 – −4.4), right hippocampal volume (B = −37.1 mm^3^, 95 % CI = −66.8 – −7.3), right dentate gyrus (B = −5.32 mm^3^, 95 % CI = −9.33 – −1.31), and right subiculum (B = −8.79 mm^3^, 95 % CI = −14.37 – −3.21), was found in the intervention group (Table 5). When adjusting for the additional demographic variables, BMI at baseline and educational level, results generally remained the same (Table 5). Within-group analyses of these significant effects revealed a significant decline for the intervention group in the right subiculum (p = 0.025). No significant VBM clusters were revealed.

**Table 5:** Intervention effects on hippocampal (subfield) volume, cortical thickness and total grey matter volume in highly fatigued patients.

Outcome measures		Intervention	Control	Treatment effect† (95 % CI)	Effect Size‡	Treatment effect† (95 % CI) – extra adjustments^₸^	Effect Size‡ – extra adjustments^₸^	% change intervention^#^	% change control^#^
**Total hippocampal volume**		*N = 32*	*N = 23*						
Total	Baseline	6688 (6 8 6)	6699 (7 0 3)						
	Follow-up	6670 (6 7 1)	6730 (7 0 1)	−52.3 (-100.3 – -4.4)*	−0.08	−51.9 (-103.7 – -0.1)*	−0.08	−0.27	0.47
Left	Baseline	3322 (3 8 4)	3286 (3 7 8)						
	Follow-up	3318 (3 7 8)	3297 (3 7 2)	−15.2 (-44.6 – 14.3)	−0.04	−19.8 (-51.1 – 11.6)	−0.05	−0.12	0.35
Right	Baseline	3366 (3 3 1)	3413 (3 5 2)						
	Follow-up	3351 (3 2 4)	3433 (3 5 1)	−37.1 (-66.8 – -7.3)*	−0.11	−31.8 (-63.7 – 0.2)	−0.09	−0.43	0.58
**GC-ML-DG**									
Left	Baseline	272 (34)	267 (32)						
	Follow-up	270 (32)	270 (32)	−4.03 (-8.74 – 0.69)	−0.12	−4.60 (-9.60 – 0.40)	−0.14	−0.72	0.92
Right	Baseline	279 (30)	288 (35)						
	Follow-up	276 (28)	291 (33)	−5.32 (-9.33 – -1.31)*	−0.17	−5.21 (-9.53 – -0.89)*	−0.16	−0.74	0.73
**CA1**									
Left	Baseline	601 (82)	613 (80)						
	Follow-up	601 (81)	614 (84)	−0.04 (-7.07 – 6.99)	0.00	−0.01 (-7.67 – 7.64)	0.00	0.12	0.08
Right	Baseline	631 (80)	664 (79)						
	Follow-up	630 (81)	668 (76)	−5.68 (-14.14 – 2.77)	−0.07	−3.64 (-12.88 – 5.61)	−0.05	−0.19	0.60
**CA2/3**									
Left	Baseline	201 (33)	197 (28)						
	Follow-up	199 (33)	199 (28)	−3.53 (-7.44 – 0.38)	−0.11	−4.05 (-8.23 – 0.12)	−0.13	−0.75	1.06
Right	Baseline	218 (32)	233 (29)						
	Follow-up	217 (31)	233 (26)	−3.61 (-7.87 – 0.65)	−0.12	−3.59 (-8.24 – 1.07)	−0.12	−0.51	0.29
**CA4**									
Left	Baseline	237 (30)	233 (29)						
	Follow-up	235 (29)	234 (28)	−2.79 (-7.19 – 1.60)	−0.10	−3.63 (-8.28 – 1.02)	−0.12	−0.57	0.71
Right	Baseline	240 (26)	249 (32)						
	Follow-up	239 (25)	250 (30)	−3.58 (-7.30 – 0.14)	−0.12	−3.36 (-7.36 – 0.64)	−0.12	−0.48	0.50
**Subiculum**									
Left	Baseline	414 (49)	417 (72)						
	Follow-up	414 (49)	418 (70)	−2.00 (-8.36 – 4.37)	−0.03	−2.70 (-9.54 – 4.15)	−0.05	−0.21	0.14
Right	Baseline	395 (44)	394 (51)						
	Follow-up	391 (46)	399 (56)	−8.79 (-14.37 – -3.21)*	−0.19	−7.04 (-12.77 – -1.30)*	−0.15	−0.98	1.18
**Tail**									
Left	Baseline	564 (99)	526 (65)						
	Follow-up	565 (98)	531 (67)	−2.79 (-12.60 – 7.01)	−0.03	−3.37 (-13.76 – 7.02)	−0.04	0.21	0.89
Right	Baseline	581 (91)	553 (67)						
	Follow-up	581 (88)	554 (71)	−1.27 (-8.53 – 6.00)	−0.02	−0.60 (-8.37 – 7.17)	−0.01	−0.08	0.30
**Cortical thickness (in mm)^**									
	Baseline	2.41 (0.07)	2.42 (0.06)						
	Follow-up	2.40 (0.08)	2.41 (0.07)	0.001 (-0.017 – 0.020)	0.02	0.002 (-0.018 – 0.022)	0.03	−0.13	−0.13
**Total grey matter (in cm^3^)^**									
	Baseline	610 (46)	617 (51)						
	Follow-up	608 (46)	613 (53)	0.92 (-4.65 – 6.48)	0.02	0.95 (-5.11 – 7.01)	0.02	−0.35	−0.58

Values indicate mean (SD) and are presented in mm^3^, unless denoted otherwise.

Abbreviations: GC-ML-DG, granule cell layer of dentate gyrus; CA, cornu ammonis.

† The intervention effect is the regression coefficient of a linear regression analysis adjusted for baseline, age, endocrine therapy and total intracranial volume calculated by CAT12.

^₸^ Analyses adjusted for body mass index at baseline and educational level, in addition to baseline measurement, age, endocrine therapy use and total intracranial volume.

‡ Effect Sizes (ES) were calculated by dividing Beta by the pooled SD at baseline, with positive ESs meaning a beneficial effect of the intervention on a specific outcome. ESs < 0.2 indicate “no difference”, ESs between 0.2 and 0.5 indicate “small differences”, ESs between 0.5 and 0.8 indicate “medium differences” and ESs ≥ 0.8 indicate “large differences” (Cohen, 2013). An ES of 0.5 or higher was considered clinically relevant (Norman et al., 2003).

^#^ Calculation for average % change between baseline and follow-up: (follow-up - baseline)/baseline * 100.

* Significant intervention effects (p < 0.05).

^ Data of one additional patient from the intervention group was missing due to segmentation errors.

For our primary cognitive outcome (HVLT-R total recall), we found that decreases in right hippocampal volume (B = −0.023, 95 % CI = −0.041 – −0.006), right dentate gyrus (B = −0.134, 95 % CI = −0.265 – −0.003) and right subiculum (B =  −0.113, 95 % CI = −0.209 – −0.018) were significantly related to an improvement in HVLT-R total recall. Moreover, decrease in total hippocampal volume (B = −0.033, 95 % CI = −0.056 – −0.009), right hippocampal volume (B = −0.041, 95 % CI = −0.081 – −0.000), and right dentate gyrus (B = −0.432, 95 % CI = −0.704 – −0.160) were significantly related to an increase in ACS Wordlist Learning performance.

## 4 Discussion

The PAM study is the first sufficiently powered randomized controlled trial designed to investigate the effects of a 6-month exercise intervention on cognitive functioning and brain structure/function in previously physically inactive breast cancers patients with cognitive problems. In the current study, we examined the effects on grey matter volumetric measures. At baseline, higher physical fitness levels were associated with larger total hippocampal volume, cortical thickness, and total grey matter volume. We observed no exercise effect on total hippocampal volume, our primary imaging outcome, and no effect on hippocampal subfield volumes, cortical thickness, and voxel-based grey matter volumes in chemotherapy-exposed breast cancer patients with cognitive problems. In highly fatigued patients, we observed an intervention effect on physical fitness of similar size. However, a significant reduction in hippocampal volume was found post-intervention, which was related to improved memory functioning. This was an unexpected finding and will be discussed below.

In animal models beneficial effects of physical exercise on brain volume have been consistently found (van Praag, 2008), while in humans these effects are observed less regularly. In a systematic review and *meta*-analysis of Firth et al. (Firth et al., 2018), including a variety of human populations, a significant effect of aerobic exercise was revealed on left hippocampal volume and Li et al. (Li et al., 2017) reported in a comparable systematic review significant exercise intervention effects in the right hippocampus. Wilckens et al. (Wilckens et al., 2021) revealed in their *meta*-analysis a positive exercise effect on total hippocampal volume. On the other hand, Hvid et al. (Hvid et al., 2021) argued in their systematic review that the effects of physical exercise on grey matter volume, including the hippocampus, are sparse and inconclusive.

Several explanations exist for the lack of intervention effects on brain volumes in our study. Wilckens et al. (Wilckens et al., 2021) reported significant exercise effects on hippocampal volume in patients aged 65 years or older, and not in participants younger than 65 years, possibly due to higher vulnerability to hippocampal atrophy in older subjects. Our sample was on average considerably younger than 65 years, which might be a reason why we did not find an exercise effect on brain volume. However, as the duration and intensity of the interventions also differed between studies in the different age groups, it is yet unknown whether age is a moderator in the association between physical exercise and brain volume, or whether intensity and duration of the intervention are in themselves explanatory factors.

Despite the lack of an effect of the physical exercise intervention on brain volumes, we did find, pre-intervention, a positive association between physical fitness levels and hippocampal volume. This kind of association has been reported previously in various populations, including breast cancer patients (Aghjayan et al., 2021, Chaddock-Heyman et al., 2015). The discrepancy between the cross-sectional positive association between fitness levels and brain measures on the one hand, and absence of interventional findings on the other hand can have several explanations. Exercise is often part of a healthier lifestyle, which might amplify physical activity effects on brain structure. Indeed, lifestyle factors including obesity, smoking and alcohol consumption are all related to lower grey matter volume (Fernández-Andújar et al., 2021, Herrmann et al., 2019, Vňuková et al., 2017, Wilson et al., 2017, Yang et al., 2020). Additionally, in rodents, a combination of voluntary physical exercise and environmental enrichment showed greater increase on hippocampal neurogenesis than either condition alone (Fabel et al., 2009, Olson et al., 2006). A physical exercise intervention per se may therefore not have been sufficient to induce measurable effects. Another explanation is that a lifetime of higher physical activity and associated life style factors, probably has a larger effect on the brain than a 6-month intervention, which would explain the positive cross-sectional findings. However, Wilckens et al. (Wilckens et al., 2021) did uncover an exercise effect for interventions longer than 24 weeks, indicating that increasing physical activity for 6 months (or more) is enough to induce volumetric changes in some populations.

In addition to environmental effects and the length of the intervention, timing of the intervention might also contribute to the intervention effect. Although we deliberately offered the intervention 2–4 years after diagnosis (when patients were post-acute and short-term cancer treatment effects, but still experienced difficulties in daily life), it is possible that the intervention might have been more effective when embedded shortly after treatment. Whether exercise during chemotherapy can contribute to prevention of (hippocampal) volume loss is also still unknown, but currently under investigation (Kiesl et al., 2022).

In our recent article covering the exercise effects on cognition, an intervention effect was described in highly fatigued patients on learning and memory functioning, and processing speed (Koevoets et al., 2022). The current study extended these findings with a significant reduction (and not an increase) post intervention in total hippocampal volume, right hippocampal volume, right dentate gyrus and right subiculum volume, which was moreover associated with improved memory functioning. The direction of this association was unexpected, although Ten Brinke et al. (Ten Brinke et al., 2015) also observed an inverse relation between hippocampal volume increase and reduced memory performance after an exercise intervention in patients with mild cognitive impairment. One could speculate that long-term inflammation after breast cancer and its treatment may result in cellular swelling and might increase brain volume first, before atrophy takes place. In agreement with this idea, even 20 years after cancer (treatment) higher inflammatory markers have been found in patients compared to controls (Van Der Willik et al., 2018) and both fatigue and cancer-related cognitive impairment have been related to (neuro)inflammation (Kurzrock, 2001, Wefel et al., 2015). Subsequently, physical exercise might have resulted in decreased brain volume, by its anti-inflammatory effects. We stress that these findings should first be replicated and future studies are encouraged to zoom in at the potential mechanisms that may underlie these effects, as clearly much is still unknown.

Hippocampal volume was thoroughly investigated, since it was examined at the subfield level. However, interpretability of the subfield segmentations is potentially limited. Resolution of the 3D T1-weighted MRI scan acquired with a 3 Tesla brain MRI scanner is considered low for (automated) hippocampal subfield segmentations (Wisse et al., 2014) and although it is not the focus of the present study, absolute hippocampal subfield volumes should be interpreted with caution. It is not uncommon to use 3 Tesla MRI scans for hippocampal subfield segmentation in FreeSurfer and intervention effects have been found using this method (e.g. for exercise (Frodl et al., 2020, Papiol et al., 2017) and electroconvulsive therapy (Cao et al., 2018)). Additionally, we did observe between-subject variation, while within-subject variation was low, and observed an inverse association between (hippocampal) volume and age. Therefore, we argue that this method can be applied to describe group differences. Another limitation is that sleep quality was not assessed. Sleep quality might be a mediator of the intervention effects in highly fatigued patients and information about sleep quality could have provided more insight in the mechanism behind the effects. We therefore suggest future research to incorporate sleep quality in their study design.

Strengths of the PAM study are the length (6 months), intensity (4 hrs/week) and type of intervention (aerobic, including high intensity interval training, and resistance training). Additionally, the intervention was partly supervised, and patients showed good adherence to the program, which subsequently resulted in improved physical fitness. To specifically target a relevant population, only relatively inactive patients who experienced and showed cognitive difficulties were selected for participation. Although the percentage of missing MRI data was relatively high due to COVID-19 measures, the current study is still one of the largest randomized controlled trials investigating the effects of physical exercise on grey matter volume.

## 5 Conclusion

A moderate-to-high intensity 6-month exercise intervention did not change total hippocampal volume, hippocampal subfield volumes, cortical thickness and voxel-based grey matter volumes in patients with affected cognition after breast cancer treatment. However, results suggested that physical exercise leads to hippocampal volume reduction in highly fatigued breast cancer patients, which was related to better memory functioning. Future research should target highly fatigued cancer patients and focus on the relation between hippocampal volume change and memory improvement.

## CRediT authorship contribution statement

**E.W. Koevoets:** Conceptualization, Methodology, Software, Formal analysis, Investigation, Data curation, Writing – original draft. **M.I. Geerlings:** Conceptualization, Methodology, Writing – review & editing, Supervision. **E.M. Monninkhof:** Conceptualization, Methodology, Writing – review & editing, Supervision, Funding acquisition. **R. Mandl:** Software, Data curation, Writing – review & editing. **L. Witlox:** Conceptualization, Investigation, Writing – review & editing. **E. van der Wall:** Conceptualization, Resources, Writing – review & editing. **M.M. Stuiver:** Conceptualization, Writing – review & editing. **G.S. Sonke:** Conceptualization, Resources, Writing – review & editing. **M.J. Velthuis:** Conceptualization, Writing – review & editing. **J.J. Jobsen:** Conceptualization, Resources, Writing – review & editing. **J. van der Palen:** Conceptualization, Writing – review & editing. **M.E.M.M. Bos:** Resources, Writing – review & editing. **E. Göker:** Resources, Writing – review & editing. **M.B.E. Menke-Pluijmers:** Resources, Writing – review & editing. **D.W. Sommeijer:** Resources, Writing – review & editing. **A.M. May:** Conceptualization, Methodology, Writing – review & editing, Supervision. **M.B. de Ruiter:** Conceptualization, Methodology, Writing – review & editing, Supervision. **S.B. Schagen:** Conceptualization, Methodology, Writing – review & editing, Supervision. **Annebeth W. Haringhuizen:** . **Wim A. van der Steeg:** . **Frederiek Terheggen:** . **Charlotte Blanken-Peeters:** . **Harold Fliervoet:** . **Margrethe S. Schlooz-Vries:** . **Tanja G. Frakking:** . **Marc W.A. van Tilburg:** . **Corina Oldenhuis:** . **Maartje F. Sier:** . **Carmen C. van der Pol:** . **Lidwine W. Tick:** . **Nel A. van Holsteijn:** .

## Declaration of Competing Interest

The authors declare that they have no known competing financial interests or personal relationships that could have appeared to influence the work reported in this paper.

## Data Availability

Data will be made available on request.
