# Supplementary material for: Effect of physical exercise on the hippocampus and global grey matter volume in breast cancer patients: A randomized controlled trial (PAM study)
Source: Neuroimage Clin. 2022 Dec 14;37:103292. doi: 10.1016/j.nicl.2022.103292 (PMC9800528; doi:10.1016/j.nicl.2022.103292)
Supplement: Supplementary data 1 [file mmc1.docx]

**Additional file**

**Table 1.** Intervention effects on hippocampal (subfield) volume, cortical thickness and total grey matter volume, per protocol.

| **Outcome measures** | | **Intervention** | **Control** | **Treatment effect† (95% CI)** | **Effect Size‡** | **% change**  **intervention** | **% change control** |
| --- | --- | --- | --- | --- | --- | --- | --- |
| **Total hippocampal volume** | | *N=52* | *N=71* |  |  |  |  |
| Total | Baseline  Follow-up | 6522 (576)  6521 (588) | 6623 (599)  6628 (604) | -7.1 (-38.5 – 24.3) | -0.01 | 0.00 | 0.07 |
| Left | Baseline  Follow-up | 3217 (322)  3218 (324) | 3275 (314)  3275 (318) | -0.1 (-20.8 – 20.6) | 0.00 | 0.05 | 0.00 |
| Right | Baseline  Follow-up | 3305 (283)  3303 (292) | 3348 (308)  3353 (307) | -8.0 (-26.0 – 10.1) | -0.03 | -0.06 | 0.14 |
| **GC-ML-DG** | |  |  |  |  |  |  |
| Left | Baseline  Follow-up | 263 (30)  262 (30) | 266 (25)  267 (26) | -2.41 (-5.35 – 0.52) | -0.09 | -0.38 | 0.38 |
| Right | Baseline  Follow-up | 274 (25)  273 (25) | 280 (30)  280 (30) | -1.23 (-3.54 – 1.08) | -0.04 | -0.21 | 0.02 |
| **CA1** | |  |  |  |  |  |  |
| Left | Baseline  Follow-up | 589 (78)  590 (78) | 609 (71)  609 (74) | 0.09 (-4.54 – 4.72) | 0.00 | 0.09 | 0.07 |
| Right | Baseline  Follow-up | 630 (70)  629 (73) | 645 (73)  647 (73) | -2.30 (-6.96 – 2.37) | -0.03 | -0.01 | 0.38 |
| **CA2/3** | |  |  |  |  |  |  |
| Left | Baseline  Follow-up | 195 (31)  195 (31) | 194 (27)  195 (27) | -1.64 (-4.25 – 0.96) | -0.06 | -0.45 | 0.32 |
| Right | Baseline  Follow-up | 219 (26)  219 (26) | 224 (30)  224 (30) | -0.66 (-3.21 – 1.89) | -0.02 | 0.01 | 0.17 |
| **CA4** | |  |  |  |  |  |  |
| Left | Baseline  Follow-up | 229 (26)  228 (27) | 232 (22)  233 (23) | -1.70 (-4.43 – 1.02) | -0.07 | -0.25 | 0.31 |
| Right | Baseline  Follow-up | 236 (21)  236 (20) | 242 (28)  242 (27) | -0.71 (-2.87 – 1.45) | -0.03 | 0.02 | 0.05 |
| **Subiculum** | |  |  |  |  |  |  |
| Left | Baseline  Follow-up | 400 (52)  401 (51) | 412 (64)  411 (61) | 1.87 (-2.65 – 6.39) | 0.03 | 0.35 | -0.30 |
| Right | Baseline  Follow-up | 379 (36)  378 (39) | 386 (50)  387 (51) | -2.03 (-6.16 – 2.10) | -0.05 | -0.31 | 0.21 |
| **Tail** | |  |  |  |  |  |  |
| Left | Baseline  Follow-up | 542 (82)  544 (82) | 541 (65)  540 (66) | 2.65 (-3.43 – 8.74) | 0.04 | 0.30 | -0.19 |
| Right | Baseline  Follow-up | 572 (100)  574 (101) | 562 (68)  562 (69) | 1.54 (-3.47 – 6.55) | 0.02 | 0.37 | 0.08 |
| **Cortical thickness (in mm)^** | | | | | | | |
|  | Baseline  Follow-up | 2.40 (0.06)  2.40 (0.07) | 2.41 (0.07)  2.41 (0.07) | -0.003 (-0.013 – 0.008) | -0.05 | 0.02 | 0.17 |
| **Total grey matter (in cm^3^)^** | | | | | | | |
|  | Baseline  Follow-up | 596 (43)  594 (42) | 599 (52)  599 (51) | -1.52 (-4.91 – 1.88) | -0.03 | -0.18 | 0.06 |

Values indicate mean (SD) and are presented in mm^3^, unless denoted otherwise.

Abbreviations: GC-ML-DG, granule cell layer of dentate gyrus; CA, cornu ammonis.

† The intervention effect is the regression coefficient of a linear regression analysis adjusted for baseline, age, endocrine therapy and total intracranial volume calculated by CAT12.

‡ Effect Sizes (ES) were calculated by dividing Beta by the pooled SD at baseline, with positive ESs meaning a beneficial effect of the intervention on a specific outcome. ESs <0.2 indicate “no difference”, ESs between 0.2 and 0.5 indicate “small differences”, ESs between 0.5 and 0.8 indicate “medium differences” and ESs ≥ 0.8 indicate “large differences” (Cohen, 2013). An ES of 0.5 or higher was considered clinically relevant (Norman et al., 2003).

^#^ Calculation for average % change between baseline and follow-up: (follow-up - baseline)/baseline * 100.

^ Data of one additional patient from the intervention group was missing due to segmentation errors.

**References**

Cohen, J., 2013. Statistical Power Analysis for the Behavioral Sciences. Academic press.

Norman, G.R., Sloan, J.A., Wyrwich, K.W., 2003. Interpretation of changes in health-related quality of life the remarkable universality of half a standard deviation. Med. Care 41, 582–592. https://doi.org/10.1097/00005650-200305000-00004
